# Supplementary material for: Improvement of Colonoscopic Image Quality Using a New LED Endoscopic System with Specialized Noise Reduction
Source: Diagnostics (Basel). 2025 Jun 19;15(12):1569. doi: 10.3390/diagnostics15121569 (PMC12192087; doi:10.3390/diagnostics15121569)
Supplement: Supplementary file 1 [file diagnostics-15-01569-s001.zip › diagnostics-3666832-supplementary/Supplementary Materials.pdf]

Supplemental Table S1. The evaluation of endoscopists of brightness and halation of cecal images for WLI, BLI, and LCI

		Group 1 EP-8000+EC-860ZP	Group 2 EP-8000+EC-760ZP	Group 3 VP-7000+EC-760ZP	P value Group 1 vs. 3	P value Group 2 vs. 3	P value Group 1 vs. 2
Brightness Overall, mean±SD	WLI	3.71±0.55	3.79±0.44	3.51±0.58	<0.001	<0.001	0.053
	BLI	3.15±0.85	2.68±0.99	2.23±0.92	<0.001	<0.001	<0.001
	LCI	3.83±0.42	3.70±0.57	3.54±0.58	<0.001	0.002	<0.001
Brightness Experts, mean±SD	WLI	3.97±0.17	3.95±0.22	3.69±0.49	<0.001	<0.001	0.236
	BLI	3.46±0.61	2.92±0.74	2.31±0.84	<0.001	<0.001	<0.001
	LCI	3.97±0.17	3.91±0.29	3.74±0.50	<0.001	0.001	0.037
Brightness Non-experts, mean±SD	WLI	3.44±0.66	3.62±0.53	3.32±0.60	0.089	<0.001	0.016
	BLI	2.83±0.94	2.45±1.06	2.14±0.98	<0.001	0.016	0.003
	LCI	3.70±0.58	3.48±0.68	3.33±0.58	<0.001	0.049	0.006
Halation Overall, mean±SD	WLI	3.60±0.51	3.45±0.57	3.18±0.59	<0.001	<0.001	0.003
	BLI	2.99±0.69	2.87±0.78	2.71±0.78	<0.001	0.018	0.031
	LCI	3.33±0.60	3.30±0.52	3.10±0.58	<0.001	0.431	<0.001
Halation Experts, mean±SD	WLI	3.83±0.38	3.63±0.49	3.33±0.60	<0.001	<0.001	<0.001
	BLI	3.45±0.52	3.25±0.56	3.13±0.53	<0.001	0.059	0.004
	LCI	3.61±0.51	3.11±0.57	3.30±0.52	<0.001	0.039	<0.001
Halation Non-experts, mean±SD	WLI	3.36±0.52	3.27±0.60	3.03±0.54	<0.001	0.001	0.129
	BLI	2.53±0.52	2.48±0.61	2.29±0.76	0.005	0.027	0.267
	LCI	3.05±0.55	2.92±0.56	3.04±0.55	0.449	0.064	0.051

WLI: white light imaging, BLI: blue light imaging, LCI: linked color imaging

Supplemental Table S2. The evaluation of image-analysis software of brightness and halation of cecal location for WLI, BLI, and LCI

Brightness	Group 1 EP-8000+EC-860ZP	Group 2 EP-8000+EC-760ZP	Group 3 VP-7000+EC-760ZP	P value Group 1 vs. 3	P value Group 2 vs. 3	P value Group 1 vs. 2
WLI, mean±SD	122.7±6.6	129.4±9.0	110.7±8.7	<0.001	<0.001	0.028
BLI, mean±SD	99.1±10.9	73.1±14.9	69.3±11.5	<0.001	0.439	<0.001
LCI, mean±SD	139.4±9.2	130.2±11.8	113.1±10.7	<0.001	<0.001	0.024
**Halation**	**Group 1 EP-8000+EC-860ZP**	**Group 2 EP-8000+EC-760ZP**	**Group 3 VP-7000+EC-760ZP**	**P value Group 1 vs. 3**	**P value Group 2 vs. 3**	**P value Group 1 vs. 2**
WLI, mean±SD	1.34±1.80	1.57±1.43	3.35±3.59	0.031	0.043	0.694
BLI, mean±SD	1.81±1.23	2.12±1.80	2.39±1.98	0.173	0.347	0.297
LCI, mean±SD	1.48±0.83	2.58±2.36	4.06±3.99	0.010	0.113	0.049

WLI: white light imaging, BLI: blue light imaging, LCI: linked color imaging, SD: standard deviation

Supplemental Table S3. The evaluation of visibility of lesions for WLI, BLI, and LCI without magnification according to lesions morphology

		Group 1 EP-8000+EC-860Z	Group 2 EP-8000+EC-760Z	Group 3 VP-7000+EC-760Z	P value Group 1 vs. 3	P value Group 2 vs. 3	P value Group 1 vs. 2
Visibility Non-polypoid, mean±SD	WLI	3.15±0.85	2.79±0.89	2.92±0.85	0.016	0.118	<0.001
	BLI	3.60±0.62	3.32±0.73	3.25±0.71	<0.001	0.221	<0.001
	LCI	3.55±0.62	3.26±0.76	3.30±0.70	0.002	0.359	<0.001
Visibility Polypoid, Mean±SD	WLI	3.41±0.69	3.39±0.67	3.30±0.72	0.142	0.200	0.420
	BLI	3.77±0.43	3.67±0.49	3.48±0.63	<0.001	0.014	0.058
	LCI	3.71±0.54	3.60±0.51	3.33±0.71	<0.001	0.001	0.079

WLI: white light imaging, BLI: blue light imaging, LCI: linked color imaging, SD: standard deviation

Supplemental Table S4. The evaluation of brightness, halation and visibility of lesions for BLI and LCI with magnification

	Overall	Group 1 EP-8000+EC-860ZP	Group 2 EP-8000+EC-760ZP	Group 3 VP-7000+EC-760ZP	P value Group 1 vs. 3	P value Group 2 vs. 3	P value Group 1 vs. 2
Brightness Overall, mean±SD	BLI	3.89±0.33	3.65±0.61	3.47±0.58	<0.001	<0.001	<0.001
	LCI	3.95±0.23	3.77±0.43	3.69±0.48	<0.001	0.034	<0.001
Brightness Experts, mean±SD	BLI	3.86±0.34	3.62±0.60	3.36±0.57	<0.001	<0.001	<0.001
	LCI	3.96±0.18	3.76±0.42	3.69±0.48	<0.001	0.014	<0.001
Brightness Non-experts, mean±SD	BLI	3.91±0.31	3.65±0.61	3.57±0.56	<0.001	0.085	<0.001
	LCI	3.92±0.25	3.76±0.42	3.74±0.48	<0.001	0.337	<0.001
Halation Overall, mean±SD	BLI	3.13±0.63	3.09±0.66	2.87±0.68	<0.001	<0.001	0.245
	LCI	3.13±0.58	3.09±0.60	2.99±0.59	0.005	0.027	0.250
Halation Experts, mean±SD	BLI	3.29±0.60	3.18±0.69	3.02±0.68	0.001	0.044	0.100
	LCI	3.25±0.59	3.14±0.69	2.96±0.66	<0.001	0.022	0.090
Halation Non-experts, mean±SD	BLI	2.97±0.60	3.00±0.62	2.71±0.63	0.001	<0.001	0.369
	LCI	3.00±0.53	3.04±0.48	3.00±0.49	0.445	0.318	0.272
Visibility Overall, mean±SD	BLI	3.83±0.40	3.75±0.48	3.49±0.58	<0.001	<0.001	0.024
	LCI	3.67±0.58	3.40±0.60	3.28±0.64	<0.001	<0.001	<0.001
Visibility Experts, mean±SD	BLI	3.92±0.29	3.51±0.64	3.44±0.58	<0.001	<0.001	<0.001
	LCI	3.75±0.57	3.51±0.64	3.15±0.60	<0.001	<0.001	0.001
Visibility Non-experts, mean±SD	BLI	3.75±0.46	3.79±0.44	3.53±0.57	0.001	<0.001	0.299
	LCI	3.59±0.57	3.46±0.54	3.40±0.64	0.010	0.209	0.037

BLI: blue light imaging, LCI: linked color imaging, SD: standard deviation

Supplemental Table S5. The diagnostic ability of images with BLI magnification using JNET classification

	8000+860 N=56	8000+760 N=62	7000+760 N=54	P value 8000+860 vs. 7000+760	P value 8000+760 vs. 7000+760	P value 8000+860 vs. 8000+760
Overall, Diagnostic accuracy, % (n)	91.5 205/224	92.3 229/248	84.6 (176/208)	0.026	0.009	0.743
Experts, Diagnostic accuracy, % (n)	92.9 104/112	92.7 115//124	85.6 89/104	0.083	0.079	0.972
Non-experts, Diagnostic accuracy, % (n)	90.2 101/112	91.9 114/124	83.7 87/104	0.153	0.053	0.635

JNET: Japan NBI expert team, BLI: blue laser imaging

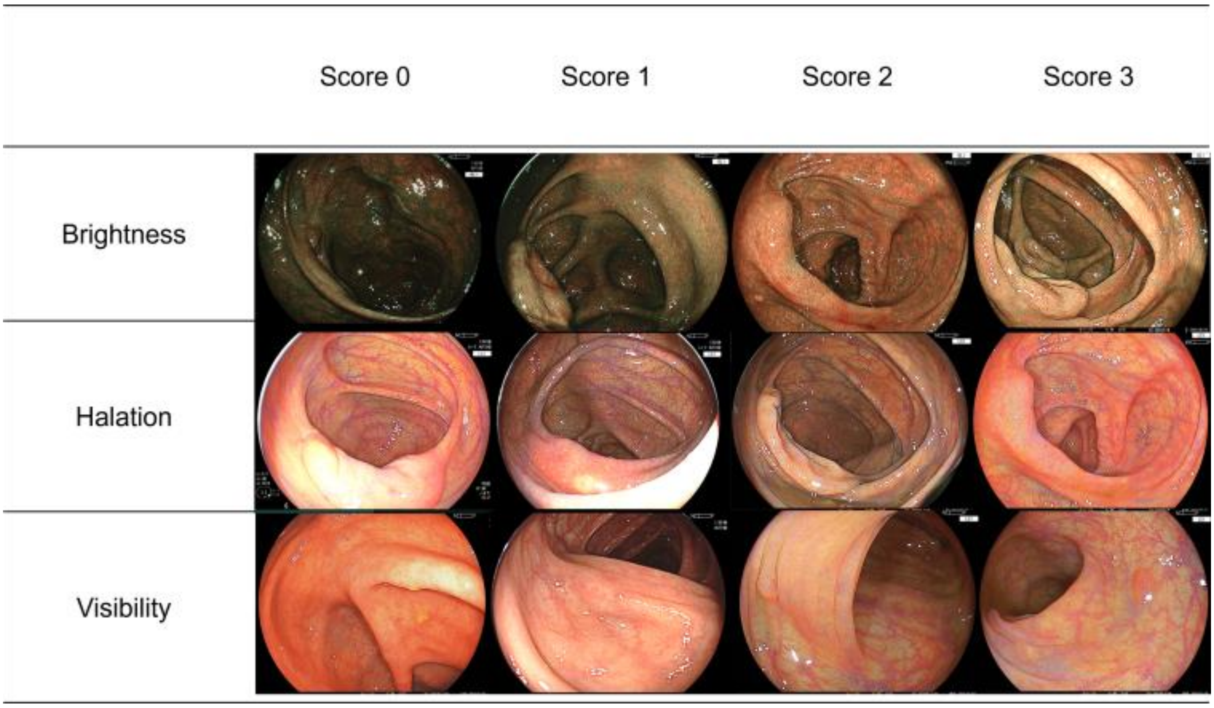

Figure S1. The evaluation of brightness, halation, and lesion visibility of cecal location and lesions. The brightness, halation and lesion visibility of cecal and lesion images (WLI, BLI, and LCI) were evaluated by endoscopists using a 4-point scale (Score 1=poor, Score 2=fair, Score 3=good, Score 4=excellent).
